# Alignment-free $d_2^*$ oligonucleotide frequency dissimilarity measure improves prediction of hosts from metagenomically-derived viral sequences

**DOI:** 10.1093/nar/gkw1002

**Published:** 2016-11-28

**Authors:** Nathan A. Ahlgren, Jie Ren, Yang Young Lu, Jed A. Fuhrman, Fengzhu Sun

**Affiliations:** 1 Department of Biological Sciences, University of Southern California, 3616 Trousdale Pkwy Los, Angeles, CA 90089, USA; 2 Molecular and Computational Biology Program, University of Southern California, 1050 Childs Way, Los Angeles, CA 90089, USA; 3 Center for Computational Systems Biology, Fudan University, Shanghai 200433, China

## Abstract

Viruses and their host genomes often share similar oligonucleotide frequency (ONF) patterns, which can be used to predict the host of a given virus by finding the host with the greatest ONF similarity. We comprehensively compared 11 ONF metrics using several *k*-mer lengths for predicting host taxonomy from among ∼32 000 prokaryotic genomes for 1427 virus isolate genomes whose true hosts are known. The background-subtracting measure $d_2^*$ at *k* = 6 gave the highest host prediction accuracy (33%, genus level) with reasonable computational times. Requiring a maximum dissimilarity score for making predictions (thresholding) and taking the consensus of the 30 most similar hosts further improved accuracy. Using a previous dataset of 820 bacteriophage and 2699 bacterial genomes, $d_2^*$ host prediction accuracies with thresholding and consensus methods (genus-level: 64%) exceeded previous Euclidian distance ONF (32%) or homology-based (22-62%) methods. When applied to metagenomically-assembled marine SUP05 viruses and the human gut virus crAssphage, $d_2^*$-based predictions overlapped (i.e. some same, some different) with the previously inferred hosts of these viruses. The extent of overlap improved when only using host genomes or metagenomic contigs from the same habitat or samples as the query viruses. The $d_2^*$ ONF method will greatly improve the characterization of novel, metagenomic viruses.

## INTRODUCTION

It is widely recognized that the ‘uncultured majority’ of bacteria and archaea (prokaryotes) dominate biomass in many ecosystem, control important global biogeochemical cycles, and significantly impact the health of humans, animals, and crops (1). Much less is known, however, about the viruses that infect bacteria and archaea. Viruses generally outnumber the abundance of prokaryotes and are estimated to represent the most abundant biological entity on the planet (2,3). They are important in limiting the abundance of their hosts, thus they can significantly impact the processes and ecosystem functions that prokaryotes carry out (4–6). For example, in marine environments viruses can be responsible for killing up to 40% of the standing stock of prokaryotes daily (6). Viruses are important mediators of evolution of their hosts. They exert strong selective pressure on host populations in the constant ‘arms race’ of virulence and resistance between viruses and their hosts. Viruses also mediate horizontal gene transfer, and thereby act as a key mediator of host genomic innovation (7).

Two basic but critical components of understanding the biology and impact of viruses on their hosts are characterizing the extant diversity of viruses and understanding what hosts different viruses infect. For many ecosystems, knowledge of these two components is still quite limited. The long-standing approach for assessing viral diversity has been isolation of new viruses using cultured host strains. The isolation approach while it also directly informs what host the virus can infect is low throughput and requires robust growth of the target host strain, which often is not possible for many bacteria and archaea (1). Virus isolation thus has vastly undersampled viral diversity (8) and the diversity of viral isolates is highly biased. Viruses have been isolated from strains that represent <15% of known phyla of bacteria and archaea (based on (9)), and of viral isolates whose genomes have been sequenced, a large portion (86%) represent those that infect only 3 of the 61 known bacterial phyla (9).

Metagenomic sequencing and assembly of viral contigs, however, provide a powerful, high throughput and culture-independent means to identify new viruses (8,10). Several recent campaigns to sequence various metaviromes at high coverage depths have significantly increased our sampling of viral diversity (11–13). While metagenomics is rapidly expanding our view of viral diversity, analysis of viral sequences assembled from metagenomes, unlike virus isolation, cannot typically reveal what host(s) they infect. With the increasing number of metagenomic datasets, there is an increasing need to predict for metagenomically-derived viruses, what hosts they likely infect.

Several computational approaches have been recently developed to discern which host(s) particular viruses likely infect based on sequence analysis. These different methods have recently been reviewed and evaluated in (14) and fall into three major categories: (i) those that use sequence homology of viruses to host genomes, (ii) co-variation analysis of viruses and hosts, and 3) sequence composition methods. The first group of methods relies on homology searches (e.g. blastn, blastx, exact short word matches) between a query virus and host genomes. Viruses and hosts can share genes or short sequence elements due to horizontal gene transfer, the sharing of short regions used in CRISPR defense systems, or integration sites used by proviruses. Homology searches are highly dependent on whether or not the host of the query virus is present in available host genome databases. Despite this, homology searches and in particular blastn produced the highest host prediction accuracy (e.g. 22–62% at the genus level) in a comparison of different methods on a benchmark dataset of 820 RefSeq virus isolate genomes and 2,699 complete bacterial host genomes (14). However, for the many novel viruses yet to be identified, homology to their potential host may be severely limited if the genomes of their respective host or related hosts have not been sequenced.

Abundance profiling and sequence composition methods differ from the aforementioned approach in that they are not dependent on sequence homology. These methods show promise in that they exhibit a strong signal for predictions, but as implemented to date they have yielded lower accuracies compared to homology methods (e.g. 25% at the genus level) (14). The abundance profiling approach infers interactions between viral and host sequences, mathematically linking those that significantly co-vary in abundance across metagenomic samples, and as such is not dependent on sequence databases. This approach was recently used to infer the host of a novel, abundant and ubiquitous viral genome found in human gut metagenomes, crAssphage (15), and to identify virus-host interactions from marine microbial metagenomes, some of which were confirmed independently by homology approaches (16). Some limitations of abundance profiling methods are that significant co-variation may not necessarily represent true infection interactions because virus-host dynamics often are complex and time-delayed and thus detection of many interactions may be missed depending on the time scale of sampling.

Sequence composition methods predict what virus a host infects based on the phenomenon that some viruses share highly similar patterns in codon usage or short nucleotide words (*k*-mers) with their hosts. Since virus replication is dependent on translational machinery of its host, selection on the virus to adopt the codons used by its hosts is thought to cause amelioration of virus nucleotide composition to that of its host (17,18). Oligonucleotide frequency (ONF) usage may also be driven by evolutionary pressure on the virus to avoid recognition sequences used by host restriction enzymes (19,20). In practice, ONF-based prediction works by computing ONF differences between a query viral sequence and a database of possible host sequences, and the predicted host is chosen as the one with the lowest ONF dissimilarity to the query (i.e. highest ONF similarity).

ONF differences between host and viral sequences have been most frequently evaluated as the Euclidean or Manhattan distance between the *k*-mer frequency vectors of virus-host pairs. Earlier studies with limited datasets of viruses and hosts show that such distance measures generally cluster together viruses and their respective hosts (20), and recent studies show that ONF measures possess informative signal for discrimination of interacting viruses and hosts (14,21). Edwards *et al*. (14) evaluated the use of Euclidean distance at various *k*-mer lengths for host prediction on 820 RefSeq bacteriophage genomes using 2,699 potential bacterial hosts with complete genomes. At *k-*mer length 4, this produced a prediction accuracy of 25% at the genus level. Roux *et al*. (21) similarly predicted host taxonomy using *k*-mer length 4 and Manhattan distance on a set of viral contigs (their methods reported using Euclidean distance but personal communication confirms actually Manhattan distance was used), but found at a much higher accuracy of >80% across the genus, family, and order levels. It is unclear if the large difference in reported accuracies between Edwards *et al*. and Roux *et al*. is caused by the different distance measures or virus and host datasets used (see Discussion), and this warrants further investigation.

While previous efforts suggest ONF methods are a feasible approach for making virus-host predictions, there is room to more fully test and improve their performance. In particular, there are several more sophisticated ONF measures that potentially could improve host prediction, but have not been evaluated. Two examples are the related measures $d_2^*$ and $d_2^S$ (22–25). These alignment-free dissimilarity measures compare two sequences based on the normalized ONFs where the expected ONFs based on a Markov model are removed from the observed ONF (see Materials and Methods section). These measures have shown excellent performance in related applications of sequence analysis—phylogenetic relatedness of genomes and congruence in sample clustering based on analysis of metagenomes and environmental conditions of those samples (22,26–28). These sophisticated dissimilarity measures have a potential advantage over simpler ONF measures like Euclidean and Manhattan distances that only use observed ONF patterns. A potential pitfall of the simple ONF approaches is that distantly related organisms potentially can evolve convergently to use similar ONF profiles. For example, while closely related bacterial genomes often share similar GC contents, distantly-related microbes can evolve independently to share similar GC contents (The streamlined genomes of marine microbes representing the phyla Cyanobacteria, Proteobacteria and Thaumarchaea, all have similar low GC contents of around 35% (29–32)). The $d_2^*$ and $d_2^S$ dissimilarity measures take into account background ONFs, that depend in large part on the GC content, and potentially can better discriminate closely related viruses or hosts by essentially comparing the two sequences on the over/under represented *k*-mers compared to expectation based on a background model (22–24). Despite their potential advantages, such background-normalizing measures have not been tested in virus-host predictions studies.

In this study, we have conducted a comprehensive evaluation of 11 oligonucleotide distance/dissimilarity measures over various *k*-mer lengths for predicting virus–host interactions. To evaluate prediction accuracy, we used a benchmark dataset of 1427 RefSeq virus isolate genomes available at the National Center for Biotechnology Information (NCBI) for which the host on which each was isolated is reported. We then computed ONF distances/dissimilarities of ∼32 000 possible bacterial and archaeal host genomes at NCBI. In addition to making predictions by simply selecting the *n* hosts with lowest dissimilarity to the query virus, we evaluated a consensus method, imposed a dissimilarity threshold for making predictions and rank-sum test approaches for picking the predicted host taxonomy. Overall the $d_2^*$ measure performed better than the other measures and exhibited stable increases in accuracy with increasing *k*-mer length. Application of the $d_2^*$ ONF method to two sets of metagenomic viral genomes produced congruent or overlapping host predictions as those previously inferred for these viruses by abundance co-variation or homology methods. In addition, we have provided a program entitled VirHostMatcher to compute ONF scores between viral and host sequences and to visualize the results.

## MATERIALS AND METHODS

### Virus and prokaryotic host databases

RefSeq genomes of viruses infecting bacteria or archaea were downloaded from NCBI on 5/8/2015. For 1427 complete viral genomes, the host on which a virus was isolated was reported under the fields ‘isolate_host =’ or ‘host =’. For initial analyses of dissimilarity measures, we used a subset of 352 viral genomes for which the isolation host was reported at the strain, subspecies, or serovar level and for which only a single host genome with that specific host name occurs in the prokaryote genome database at NCBI. (Hosts reported at the strain or serovar level were identified as those that had one of the following word formats: *Genus species* strain_name, *Genus* sp. strain_name, *Genus species* serovar serovar_name). This smaller dataset therefore consisted of 352 pairs of viral genomes and the genome of the specific host on which they were isolated. The taxonomy of the hosts on which the 1427 viruses were isolated was collected from NCBI. Host predictions were made using a database of 31 986 complete and draft bacterial and archaeal genomes downloaded from NCBI on 5/5/2015, and their taxonomies were also collected. The accession numbers and taxonomies for viral and host genomes used are provided in the supplemental table, Supplemental_table_virus_and_host_genomes.xlsx. The viral contigs and host genomes and their associated taxonomies used previously in Roux *et al*. (21) were made available by the authors and downloaded from iPlant at http://mirrors.iplantcollaborative.org/download/iplant/home/shared/ivirus/VirSorter_curated_dataset/. SUP05 virus genomes assembled from metagenomes from hydrothermal vent plume samples (33) and the crAssphage genome assembled from human gut metagenomes (15) were downloaded from the NCBI files linked to those publications. For analysis of SUP05 viruses, the set of all contigs assembled from the Guaymas basin plume metagenomes were downloaded from http://www.earth.lsa.umich.edu/geomicrobiology/data/Guaymas_454_assembly.contigs.fasta. SUP05 viral contigs and contigs shorter than 5 kb were removed, leaving 501 contigs. Except for specific analyses to test the dependence of prediction accuracy on sequence length or simulated sequencing errors. *k*-mer frequencies were determined using all nucleotides in each complete genome or all contigs within each genome sequencing project. To test the dependence of host prediction on sequencing errors, contigs were randomly subsampled from genome projects and errors were introduced at different rates by randomly making nucleotide substitutions at a probability equal to the particular error rate tested.

For some analyses, the viral isolates and host genomes were restricted to those that primarily are found in marine habitats. NCBI genome files frequently do not indicate the source habitat from which viruses or hosts were isolated, so habitat information curated for microbial genomes at Integrated Microbial Genomes (IMG, img.jgi.doe.gov) was used to generate a list of genera that were isolated from marine habitats. This list was used to select members from the 31 986 host genomes that belong to those marine genera. We further removed host genomes for particular species that are primarily found in non-marine habitats such as *Pseudomonas aeruginosa* and *Bacillus subtilis*.

### Oligonucleotide frequency measures and availability of VirHostMatcher software

Several dissimilarity measures based on genomic oligonucleotide frequencies have been developed to infer the relationship between genomic sequences. Here, we study a collection of 11 dissimilarity measures based on two different principles—those that consider background frequencies of *k*-mers and those that do not. Details (including the underlying mathematical equations) of the measures tested are provided in the Supplementary Material available online at NAR, including supplemental references for distance measures. We briefly describe the different measures here. For measures that do not consider background *k*-mer frequencies, we used several common methods for computing the distance between two vectors, in this case observed *k*-mer frequencies of each pair of host and viral sequences: Euclidean distance (*Eu*), Manhattan distance (*Ma*), Chebyshev distance (*Ch*), ${d_2}$ (34) and Jensen-Shannon divergence (*JS*) (35). The background normalization methods, including $d_2^*$, $d_2^S$ (23), *Hao* (36,37)*, Teeling* (38)*, EuF* (20) and *Willner* (39), incorporate different forms of sequence background models to compute the divergence between the observed and expected *k*-mer frequencies to eliminate the effect of the background average *k*-mer counts and enhance the signal of differences between the host and viral sequences.

We have made available a program entitled VirHostMatcher at github.com/jessieren/VirHostMatcher to compute all of the above measures and to visualize the results. The visualization tool allows users to examine the taxonomy of predicted hosts, and includes the ability to view consensus results of the top *n* most similar hosts with or without imposing a score threshold.

### Determination of predicted host taxonomy

A few methods were implemented to select the predicted host of a query virus based on pairwise distance/dissimilarity measures of the viral sequence to all possible host sequences. The simplest was to select the host with the lowest distance/dissimilarity to the query virus (i.e. greatest similarity). Alternatively a consensus method was taken. The hosts with the *n* lowest distances/dissimilarities to the query virus were taken, and the predicted taxonomy was selected as most frequent taxon at a given taxonomic level, whereby ties were chosen randomly. The prediction accuracy was calculated as the percentage of viruses whose predicted hosts had the same taxonomy as their respective known hosts, evaluated for each taxonomic level. We also used the Wilcoxon rank-sum test to determine if the hosts of a particular taxon (e.g. Proteobacteria at the phylum level) have significantly lower dissimilarity scores compared to all other taxa at that level (e.g. all other phyla). If the Bonferroni corrected *p*-value for the taxon tested was sufficiently small at the 5% significance level, this taxon was predicted as a potential host of the query virus.

### Availability of data

All genome sequences used in our analyses are publically available online via NCBI, iPlant, or www.earth.lsa.umich.edu as stated above. Relevant accession numbers for virus and host genomes are found in the supplemental file, Supplemental_table_virus_and_host_genomes.xlsx.

## RESULTS

### Initial testing of different ONF based measures

**Figure 1.**
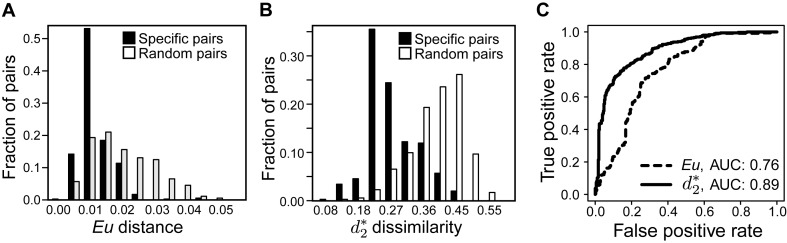
Distributions of virus-host distances/dissimilarities and ROC curves for the *Eu* and $d_2^*$ measures for *k*-mer length 6. (**A** and **B)** Virus-host distances/dissimilarities for 352 complete RefSeq virus genomes and the respective genomes of the host strains on which they were isolated (specific pairs) or 352 randomly selected pairs of the 352 RefSeq viruses and hosts (random pairs). Note that decreasing *Eu* distances and $d_2^*$ dissimilarities indicate greater virus-host similarity. (**C**) ROC curves and the corresponding area under the curve (AUC) for the *Eu* and $d_2^*$ measures. A true positive is when the virus-host pair with the lowest distance/dissimilarity predicts the true host on which the virus was isolated. (ROC curves for all measures and *k*-mer lengths 4, 6 and 9 are shown in Supplementary Figure S2).

We tested the utility of 11 different ONF distance/dissimilarity measures which belong to two major classes: those that simply use observed ONFs—*Eu, Ma, Ch*, ${d_2}$, and *JS*—and those that take into account background k-mer frequencies of the host and virus—$d_2^*$, $d_2^S$, *Hao, Teeling, EuF* and *Willner*. We first used a highly specific set of 352 genomes of viruses and the hosts on which they were isolated (see Materials and Methods section). To initially survey the discriminatory power and specificity of ONF distance/dissimilarity measures for virus-host prediction, histograms of ONF distance/dissimilarities, over several *k*-mer lengths, for the known 352 virus-host pairs were compared to 352 randomly selected virus-host pairs to simulate non-interacting pairs. The random simulation is reasonable as most of virus-host pairs should not interact. To model the background of the sequences, we used Bayesian information criterion (BIC) to estimate the MC order for the 352 phage sequences as in (35). About 70% of the phage sequences have an estimated MC order of 2. Thus, the second order MC was used as the background model. For the *Eu* and $d_2^*$ measures at *k*-mer length 6, the means of the distances/dissimilarities of known virus-host pairs were significantly different from that of the random virus-host pair (*P*-value << 0.001, *t*-test, Table 1, Figure 1A and B), supporting the potential of ONF based measures to predict true interacting virus-host pairs. The corresponding histograms for other measures and *k*-mer lengths are shown in Supplementary Figure S1. Of the 11 measures, $d_2^*$ notably had the smallest *p*-value, indicating it has the strongest potential for best predicting virus-host interactions (Table 1, Supplementary Figure S1).

**Table 1.** *t*-test *p*-values for comparisons of distance/dissimilarities of 352 virus-host pairs vs. 352 random virus-host pairs

Eu	Ma	Ch	${d_2}$	JS	$d_2^{\rm{*}}$	$d_2^S$	Hao	Teeling	EuF
2.61 × 10^−37^	5.00 × 10^−47^	9.78 × 10^−07^	2.66 × 10^−45^	4.13 × 10^−47^	**9.33 × 10^−92^**	3.55 × 10^−81^	1.37 × 10^−72^	1.82 × 10^−83^	0.997

Oligonucleotide frequency measures were computed using *k*-mer length 6 and second order MC.

To better assess the discriminatory power of the 11 dissimilarity measures, receiver operating characteristic (ROC) curves were constructed using each measure for various *k*-mer lengths. Incremental *k-*mer lengths from *k =* 4 to 9 were tested, but for simplicity we have reported results for 4, 6, 9 throughout this study. ROC curves are commonly used to visualize the performance of predicting true pairs by showing the true positive rate against the false positive rate at various thresholds (Figure 1C, Supplementary Figure S2). The resulting areas under the curve (AUC) of ROC curves provides a quantitative measure of predictive performance for the various methods, with higher values indicating higher power in discriminating true and random pairs. Among the dissimilarity measures without background correction, *JS*, ${d_2}$ and *Ma* perform similarly with AUC scores ranging from 0.81 to 0.83 (Table 2). They outperformed the widely used Euclidean (*Eu*) distance measure in predicting virus host taxonomy. Among the dissimilarity measures with background correction, $d_2^*$ generally performed the best followed by $d_2^S$ and *Teeling* that can be as high as 0.89 and 0.90 when the *k*-mer length is 6 and 9, respectively.

**Table 2.** Area under the curve (AUC) for receiver operating characteristic (ROC) curves for 352 virus-host pairs

		Length 4	Length 6	Length 9
Measures without background normalization	*Eu*	0.78	0.76	0.72
	*Ma*	0.80	0.81	**0.83**
	*Ch*	0.70	0.61	0.54
	${d_2}$	0.81	**0.82**	**0.83**
	*JS*^a^	**0.82**	**0.82**	0.82
Measures with background normalization	$d_2^*$	0.84	**0.89**	**0.90**
	$d_2^S$	0.81	0.88	0.87
	*Hao*	0.81	0.85	0.74
	*Teeling*	**0.85**	0.87	0.83
	*EuF*	0.53	0.40	0.35
	*Willner*^b^	0.63	NA	NA

The measures with the highest AUC value within each group of measures and for each *k*-mer length are listed in bold.

^a^The *JS* measure is independent of *k*-mer length.

^b^The *Willner* measure is not defined for *k*-mer lengths >4.

### Accuracy of ONF measures to predict host taxonomy

Following this initial performance test, we assessed the ability of the 11 measures to predict the correct host taxonomy for the complete set of RefSeq 1427 virus isolate genomes, for which their host is known, from among 31,986 possible bacterial and archaea genomes downloaded from NCBI. Pairwise ONF distances/dissimilarities were computed between all virus and host genomes, and the host with the lowest distance/dissimilarity score (i.e. highest similarity) to the query virus was selected as the predicted host. Prediction accuracy was scored for each level of taxonomy using *k*-mer lengths 4–9. For simplicity, results for *k*-mer length of 4, 6 and 9 are reported (Table 3). At *k*-mer length 4 (tetranucleotide frequency), *Ma* distance yielded the highest accuracy at several taxonomic levels (family, order, class, and phylum). At the genus level, $d_2^*$, *Hao* and *Teeling* outperformed *Ma*, and at the domain level *Ma* was tied with the best accuracy with four other measures—${d_2}$, *Hao, Teeling* and *EuF*—at 97%.

**Table 3.** Host prediction accuracies for 1427 viruses from among ∼32 000 prokaryotic hosts for various ONF measures

	Measures without background normalization					Measures with background normalization					
Taxon level	*Eu*	*Ma*	*Ch*	${d_2}$	*JS*^b^	$d_2^*$	$d_2^S$	*Hao*	*Teeeling*	*EuF*	*Willner*^a^
	*k*-mer length 4										
Genus	17	19	14	18	19	**21**	19	**21**	**21**	20	15
Family	29	**33**	22	29	29	32	28	31	31	32	21
Order	38	**41**	32	39	37	37	**41**	37	38	38	27
Class	58	**61**	53	59	57	52	50	54	54	52	40
Phylum	61	**65**	57	62	61	59	55	60	61	58	53
Domain	96	**97**	95	**97**	96	96	96	**97**	**97**	**97**	95
	*k*-mer length 6										
Genus	22	24	14	23	19	**33**	29	24	29	30	NA
Family	33	38	21	34	29	**48**	41	34	39	43	NA
Order	45	46	29	46	37	**54**	44	39	45	48	NA
Class	64	66	50	66	57	**67**	66	56	61	63	NA
Phylum	68	69	57	69	61	**75**	64	64	71	71	NA
Domain	**98**	**98**	95	**98**	96	97	95	96	97	96	NA
	*k*-mer length 9										
Genus	25	7	7	29	19	**44**	8	21	29	21	NA
Family	37	11	11	38	29	**57**	13	23	34	27	NA
Order	45	15	15	46	37	**62**	24	25	38	32	NA
Class	67	33	33	69	57	**73**	50	35	64	50	NA
Phylum	72	45	45	73	61	**79**	56	39	71	56	NA
Domain	98	91	91	98	96	**99**	97	95	96	96	NA

^a^NA = not applicable. The *Willner* method is not defined for *k*-mer length > 4.

^b^*JS* is defined only on MC order, regardless of the *k*-mer length. It has the same accuracy over all values of *k*.

The predicted host was selected as the host genome with the lowest ONF distance/dissimilarity to the query virus. Numbers in bold indicate, for each taxonomic level, the measure with the highest accuracy. Predictions were made for all viruses (no dissimilarity threshold was applied, see below).

**Figure 2.**
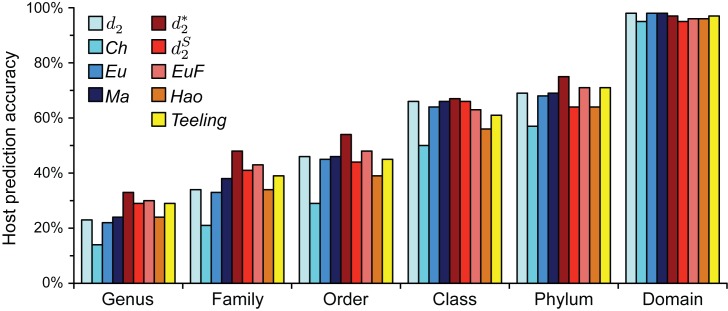
Prediction accuracy using ONF with various distance/dissimilarity measures at *k*-mer length 6 on a benchmark dataset of 1427 complete viral RefSeq genomes whose hosts are known versus ∼32,000 possible archaea and bacteria host genomes. Predictions were made for all 1427 viruses (no dissimilarity threshold was applied, see below).

Accuracy generally increased for most measures when *k*-mer length was increased to 6. Except for *Ch* and *JS*, prediction accuracy improved at all taxonomic levels except the domain level where accuracy decreased nominally for some measures. At this *k*-mer length, the background corrected methods in general performed better than non-corrected methods. For example, genus level accuracies ranged from 24% to 33% for corrected methods versus 14% to 24% for non-corrected methods. Among the background corrected methods, $d_2^*$ had the best performance over all other measures at every taxonomic level except for the domain level where ${d_2}$, *Eu* and *Ma* had only slightly higher accuracies (98% versus 97%) (Table 3, Figure 2). As *k*-mer length was increased further to 9, $d_2^*$ was the only measure that increased in accuracy at all taxonomic levels. Furthermore $d_2^*$ had the best accuracy of all measures at all taxonomic levels except for the domain level; it was tied with ${d_2}$ at 99%. Increasing the *k-*mer length to 9 also increased accuracy for ${d_2}$ and *Eu* at most taxonomic levels, the exceptions being no change at the order and domain levels. For the remaining measures, however, accuracy generally decreased. Overall, $d_2^*$ had superior performance over the other measures at higher *k*-mer lengths and exhibited consistent increases in performance with increasing *k*-mer length. In particular at *k*-mer lengths 6 and 9, $d_2^*$ had increased accuracies of 8–19% over previously used *Eu* and *Ma* measures at the lower genus, family, and order levels. This measure was used in subsequent optimizations and applications. A *k*-mer length of 6 was selected because while *k*-mer length 9 accuracies were somewhat better, they came at a high, 64-fold increase in computational time.

All prediction accuracy results in Table 3 were significantly higher than the accuracies of selecting a host at random except for at the domain level (*P*-value = 0.05, Supplementary Table S1). Because the distribution of taxa among the possible ∼32 000 hosts was very unbalanced, the accuracy for selecting a random host at each taxonomic level was empirically calculated. In general, the host prediction accuracy by choosing a random host increased with increasing taxonomic level (from genus to domain) again due to the unbalanced nature of the virus and host databases. For example at the domain level, the accuracy for picking the correct host is very high (95%) because the databases are dominated by bacteria and bacterial viruses. When comparing ONF prediction results, this means that the fold increase in prediction accuracy with over random selection increased with decreasing taxonomic level (from phylum to genus) (Supplementary Figure S3).

**Figure 3.**
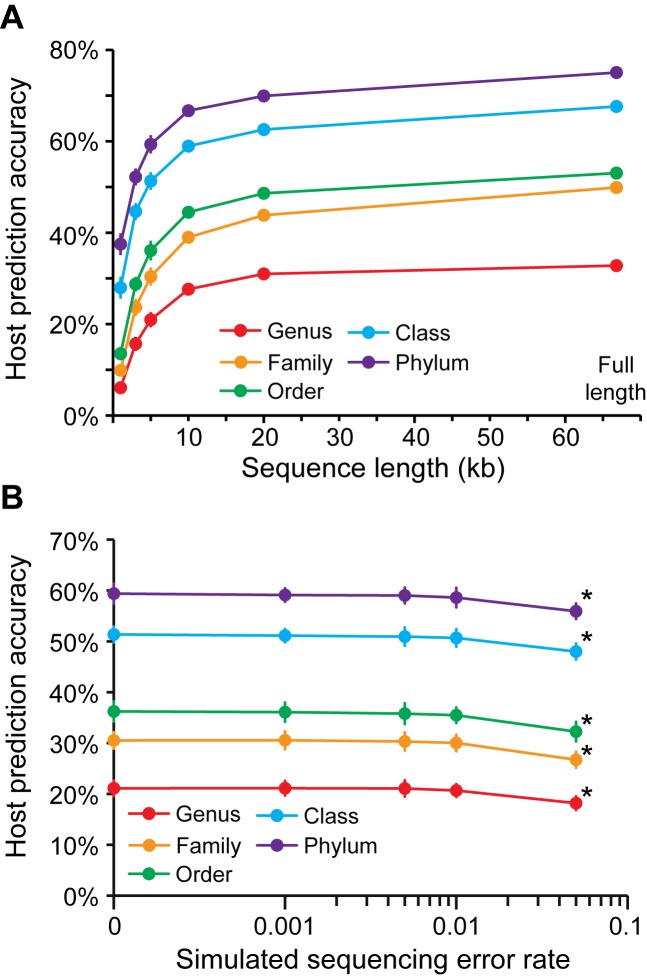
The dependence of host prediction accuracy on the length of the query viral sequence (**A**) and with simulated sequencing error (**B**). (A) Each of the 1427 complete NCBI virus genomes were randomly subsampled 30 times for several lengths. Hosts were predicted on each subsampling replicate from among ∼32 000 as the one with the lowest $d_2^*$ dissimilarity score (*k* = 6). Points depict the average of the resulting accuracies for the 1427 viruses at each taxonomic level and subsampling length. The error bars depict the 95% confidence intervals. The data points for prediction accuracies using the full length viral genomes were plotted at 66.8 kb, the mean length of the 1427 viruses (standard deviation: 54 kb). (B) Query viruses were sampled at 5 kb as above (*n* = 30), random sequencing error was simulated for these contigs at several error rates, and predictions were made on these viral contigs. Points represent the average prediction accuracy for all replicate contigs and error bars depict 95% confidence intervals two times the standard deviation. Only at an error rate of 0.05 were the prediction accuracies significantly different (*P* < 0.05, indicated with ‘*’) than no simulation of sequencing error (rate = 0). No thresholds were applied and predictions were made for all viruses.

We also tested the dependence of host prediction accuracy on the size of the query virus sequence and sequencing errors. The 1427 viral isolate genomes were subsampled at several lengths (30 replicates) and host prediction accuracy was assessed when picking the predicted host with the lowest dissimilarity $d_2^*$ score (*k* = 6) from among the ∼32 000 possible host genomes. Prediction accuracy generally increased with contig length (Figure 3A). Accuracy was relatively stable down to 10 kb with absolute decreases in accuracy of only 5–11% compared to full length sequences across the different taxonomic levels. Accuracy dropped more appreciably at shorter subsampled contig lengths of ≤ 5 kb. To test the sensitivity of this method to sequencing errors, we similarly subsampled 5 kb contigs (30 replicates) for each of the 1427 query viruses, simulated random sequencing errors on those contigs at several error rates, and made host predictions using $d_2^*$ (Figure 3B). Prediction accuracy only decreased significantly (*P*-value < 0.05 for *t*-tests) when the sequencing error rate was 0.05. For reference, the error rates of Illumina and 454 platforms are ∼0.001 or 0.01, respectively (40), so sequencing errors do not grossly impact host prediction at the reported error rates for the next-generation sequencing technologies.

### Approaches for further increasing accuracy

**Figure 4.**
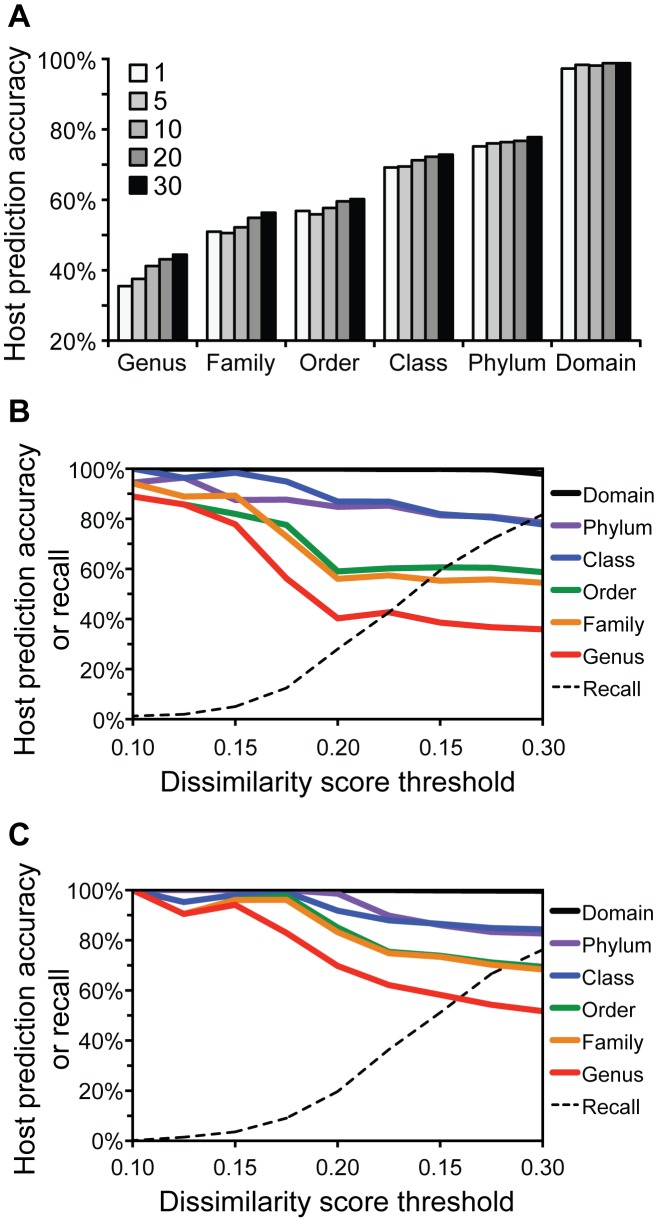
Approaches for increasing host prediction accuracy in application of the $d_2^*$ measure (*k*-mer length 6). (**A**) Prediction accuracy when using the most similar host (*n* = 1) or a consensus method whereby the predicted host is the most frequent taxon among the *n* hosts with the lowest dissimilarity scores to the query virus (*n* = 5, 10, 20, 30). (**B**) Host prediction accuracy when requiring that for a prediction to be made, the host with the lowest dissimilarity score not exceed a given threshold. (**C**) Host prediction when applying the consensus rule for *n* = 30 as in A and imposing thresholding as in B). Dissimilarities were computed using the measure, *k*-mer length 6 on 1427 RefSeq viruses and the ∼32 000 possible bacterial and archaeal host genomes. The dashed line depicts the fraction of viruses for which predictions were made given the threshold requirement (recall).

Even when using the best measure $d_2^*$ at higher *k*-mer lengths, host prediction accuracies at most taxonomic levels were often relatively low (often below 80%), so we explored approaches for improving prediction accuracy. Rather than simply selecting the predicted host as the one that had the lowest ONF distance/dissimilarity to the query virus, a maximum consensus method was applied. The predicted host taxon was selected as the most frequent taxon among the *n* hosts with the lowest distances/dissimilarities to the query virus. Using $d_2^*$ at *k*-mer length 6, prediction accuracy generally increased at all taxonomic levels using this consensus approach for *n* < 30 (Figure 4A), but at values >30, accuracy began to diminish (data not shown). The relative increase in prediction accuracy was higher for lower taxonomic levels, i.e. the genus level saw the greatest increase in performance.

We also tested applying a distance/dissimilarity threshold requirement for making host predictions, following a similar approach used in (21). Predictions were only made when the dissimilarity score of the most similar host to the query virus was smaller than a given threshold. This threshold requirement appreciably increased accuracy (Figure 4B). Thresholding however came at the cost of the number of viruses for which predictions could be made (recall), as a relatively small portion of virus-host pairs have very low ONF dissimilarities. For example, applying threshold of 0.15 could increase accuracies to above 80% at all taxonomic levels, but predictions were only made for 5% of the viruses. Combining the consensus approach with thresholding allowed for increased prediction accuracy while retaining more viruses for which a prediction could be made (Figure 4C). Using consensus prediction for the 30 most similar hosts resulting in >80% accuracy across all taxonomic levels at a higher threshold, 0.175, and increased the number of viruses for which a prediction could be made (9%).

**Figure 5.**
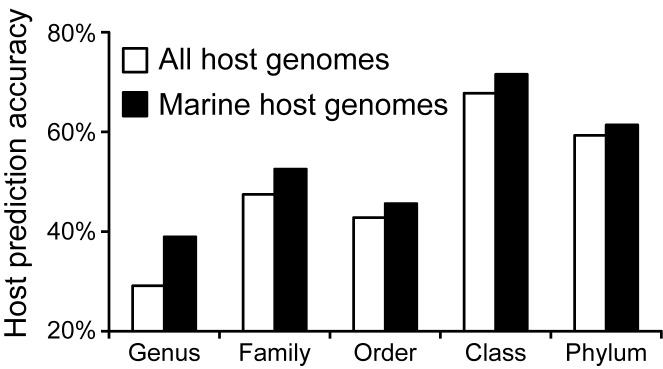
Comparison of host taxonomy prediction for 285 marine viruses when using all host genomes (*n* = 31 986) or only marine host genomes (*n* = 3277) using the measure $d_2^*$ (*k-*mer length 6). No dissimilarity threshold was applied and predictions were made for all 285 viruses.

The ∼32 000 genomes in the host database represent microbes that are found in a variety of habitats, such as soil or aquatic habitats or associated with human or animal hosts. Restricting the possible hosts used in ONF analysis to those that are found in the respective habitat of the query virus could potentially increase the accuracy of prediction. To test this, host predictions were made on 285 marine viruses in the 1427 RefSeq viruses against 3,529 possible marine hosts in the collection of 31,986 hosts from NCBI (see Methods). Having established that the $d_2^*$ measure is the best performing measure, we investigated if and how much the host prediction accuracy would be increased by focusing on marine hosts only versus all the available hosts. Figure 5 shows that host prediction accuracy is increased across all taxonomic levels when restricting the possible hosts to marine host genomes (Figure 5). Accuracy improved the most at lower taxonomic levels.

### Comparison of our improved ONF method to previous virus prediction studies

**Figure 6.**
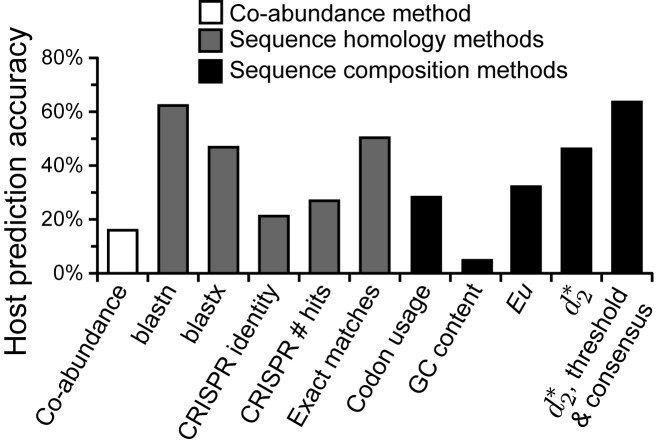
Comparison of genus level host prediction on 820 complete RefSeq virusesand 2,699 complete bacterial host genomes using different types of methods: co-abundance method (white bar); homology searches of viruses to host genomes (grey bars); and sequence composition methods (black bars), including codon, usage, virus-host similarity methods using Eu and $d_2^*$ oligonucleotide similarity measures (k-mer length 6). All results except $d_2^*$ results are as reported in Edwards *et al.* 2015. Results using the $d_2^*$ method are shown when selecting the most similar host and when requiring a score threshold of ≤ 0.25 and taking the consensus of the top five most similar hosts. The fraction of viruses for which predictions could be made with this threshold requirement was 49%.

Edwards *et al*. 2015 presented a comprehensive comparison of different methods for host prediction including virus-host abundance covariation, sequence homology to host genomes, analysis of CRISPR sequences, and ONF methods. They tested 820 bacterial viruses for which their host was reported and made host predictions using 2,699 possible complete bacterial genomes using Euclidean distance and various *k*-mer lengths. Use of the $d_2^*$ measure on this same dataset had higher prediction accuracy over *Eu* at *k*-mer length 6, improving accuracy by 25–99% across the different taxon levels (Table 4, Figure 6). The $d_2^*$ measure yielded accuracies that were comparable to those achieved by sequence homology methods (blastn, blastx, CRISPR, exact matches; Table 4, Figure 6). Applying thresholding (predictions made only on hosts with scores ≤ 0.25) and consensus approach (consensus of the top five most similar hosts), further increased prediction accuracy to levels exceeding or nearly matching all other methods. Thresholding however did come with a reduction in the number of viruses for which a prediction could be made (49%).

**Table 4.** Host prediction accuracies for 820 viruses from 2,699 host genomes from reference (14)

	Species	Genus	Family	Order	Class	Phylum
Co-abundance^a^	12	16	22	27	39	53
blastn^a^	**45**	62	74	81	89	**96**
blastx^a^	33	47	56	67	80	93
CRISPR identity^a^	15	21	31	36	47	67
CRISPR # hits^a^	22	27	37	44	52	63
Exact matches^a^	40	50	57	61	69	80
*Eu* (*k* = 6)^a,b^	13	32	41	46	59	65
$d_2^*$ (*k* = 6)^b^	26	46	59	66	73	80
$d_2^*$ (*k* = 6), score thresholding and consensus of top 5 most similar hosts^c^	39	**64**	**81**	**84**	**90**	92

Previous results reported in (14) are listed along with results from this study using $d_2^*$ (*k*-mer length 6). The highest accuracies among methods for each taxonomic level are in bold.

^a^Accuracies previously reported in Edwards *et al*. 2015.

^b^No dissimilarity threshold was applied.

^c^Predictions were only made on virus-host pairs with scores ≤0.25 (*n* = 399) and taking maximum consensus of the top 5 most similar hosts. Under this requirement, predictions were made for 49% of the viruses.

**Figure 7.**
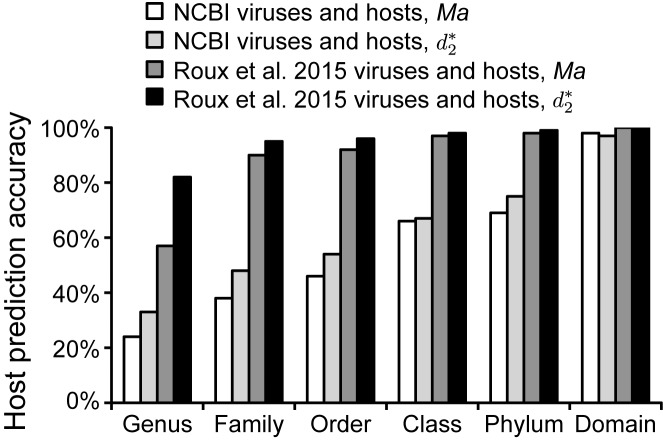
Comparison of host prediction accuracy using the similarity measures $d_2^*$ and *Ma* (*k*-mer length 6) on 1427 viral isolate genomes and ∼32 000 host genomes from NCBI (NCBI viruses and hosts) or the Roux *et al*. 2015 dataset of 12 498 viruses recovered from 14 977 host genome sequencing projects using VirSorter (Roux *et al*. viruses and hosts). Predictions were made for all viruses (no dissimilarity threshold was applied).

We noted that host prediction accuracies with the datasets of 1427 or 820 virus isolates were notably lower than results by Roux *et al*. (21) who reported genus level prediction accuracies above 80% for their set of 12 498 viral sequences (without a threshold requirement). These viral sequences were identified from within 14 977 bacterial and archaeal host genomes using their recently developed program VirSorter (9). Their host predictions were made using Manhattan (*Ma*) distance at *k*-mer length 4 and selecting the most similar host from among the 14 977 host genomes (excluding the source genome from which the virus was recovered). We did a comparable analysis of prediction accuracy with *Ma* and $d_2^*$ at *k*-mer length 6 on this same sequence dataset. $d_2^*$ had superior performance on the Roux *et al*. dataset over *Ma*, consistent with results above (Figure 7, Supplementary Table S2). Notably, the 1427 RefSeq dataset yielded appreciably lower prediction accuracy than the Roux *et al*. dataset, regardless of ONF measure used. In particular, at the lower taxonomic levels of genus, family, and order, accuracies on the Roux *et al.* dataset were roughly 2-fold higher (Figure 7).

### Differences in ONF virus-host similarity and host prediction among viral groups

**Figure 8.**
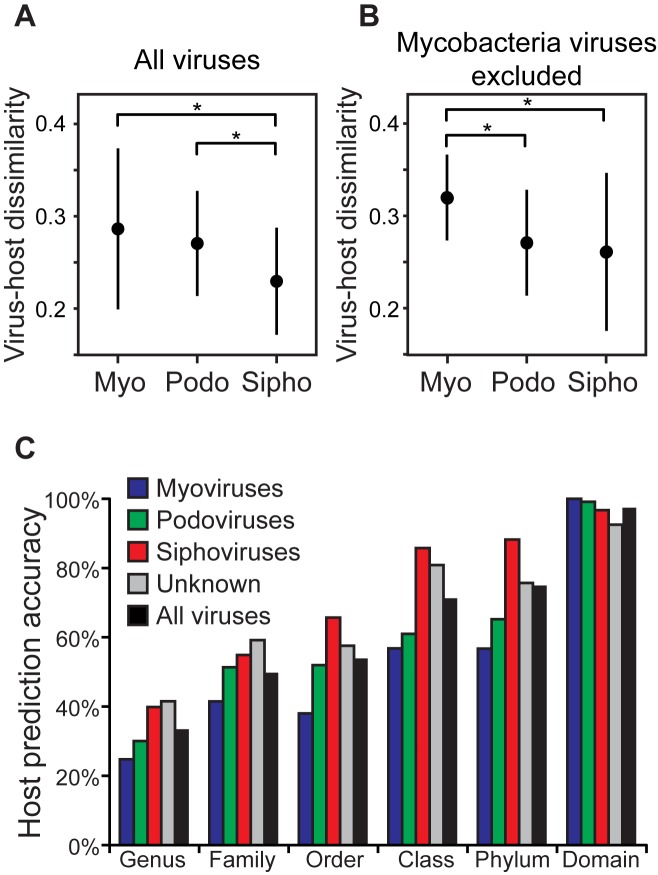
Differences in virus-host dissimilarities and prediction accuracy between the three major groups within Caudoviruses: myoviruses (Myo), podoviruses (Podo) and siphoviruses (Sipho) using the measure $d_2^*$ (*k*-mer length 6). (**A**) Virus-host dissimilarities for all caudoviruses (*n* = 332) in the 352 virus dataset and their respective hosts. (**B**) Because the taxonomy of the hosts on which these viruses were isolated is dominated by myoviruses infecting a single mycobacterium strain, results were also shown when excluding those mycobacterium viruses. In A and B, horizontal bars represent median values, boxes outline first and third quartiles, and whiskers depict 95% confidence intervals. Brackets indicate which distributions are significantly different (*t*-test, *P* < 0.001). (**C**) Host prediction accuracies for 1427 RefSeq virus genomes with ∼32 000 possible subject host genomes using $d_2^*$ and *k*-mer length 6. Results shown for all viruses; only myoviruses, podoviruses or siphoviruses; and for viruses for which their taxonomy is not reported (*n* = 109). Predictions were made for all viruses (no dissimilarity threshold was applied).

Tailed DNA viruses of the order Caudovirales are comprised of three major groups, the myoviruses, podoviruses, and siphoviruses. These groups generally exhibit different ranges in specific hosts they can infect (host range) with myoviruses often having the broadest host ranges and podoviruses and siphoviruses typically having relatively narrow host ranges (41,42). It has been suggested that such differences in host range may correspond to detectible differences in ONF virus-host similarities between these groups, with myoviruses predicted to have more relaxed similarity to their hosts. We tested this hypothesis for the 352 virus dataset which were mostly Caudoviruses (94%). Indeed, myoviruses had the highest virus-host dissimilarities (Figure 8A). Siphoviruses, which have been seen to have the most restrictive host range (41), had significantly lower dissimilarities to their hosts than myoviruses and podoviruses (*P* < 0.001, Figure 8A). When excluding siphoviruses infecting *Mycobacterium smegmatis* MC2 155 that dominated the dataset (*n* = 204), the relative order of mean virus-host dissimilarities among the virus groups remained the same (myoviruses > podoviruses > siphoviruses), but the significance of differences in distributions between groups changed somewhat (Figure 8B). Consistent with these patterns, prediction accuracies between the three different groups of caudoviruses were different (Figure 8C). Siphoviruses had notably higher accuracies than the other two caudoviral groups and when considering all viruses in the dataset, and myoviruses had the lowest prediction results.

### Testing our improved ONF methods on previous virus-host interaction studies

We tested the use of the $d_2^*$ measure in host prediction of two sets of metagenomic viruses, SUP05 group viruses and crAssphage, for which their host has been inferred by homology to host genomes and abundance profiling, respectively. The SUP05 viruses were assembled from marine hydrothermal vent plume samples and were inferred to infect the SUP05 clade of gamma Proteobacteria, an important group of sulfur oxidizers in marine ecosystems (43,44). Only a few cultured representatives of SUP05 bacteria have been recently obtained (45,46), and there are no viral isolates for this group. Metagenomic assembly of plume water samples recently yielded four discrete viral genomic ‘bins’ based on tetranucleotide composition and contig abundance (33). Fifteen of 18 genomes in those four virus bins possessed sulfur oxidation genes that were phylogenetically similar to SUP05 host genes, permitting the conclusion that these genomes represent viruses that infect SUP05 bacteria. Viruses often carry such metabolic genes obtained by horizontal gene transfer from their hosts that bolster host metabolism during infection and increase virus production (7,47). We computed $d_2^*$ similarities between these 18 viral genomes and 3,281 available marine host genomes including the two nearly complete SUP05 genomes assembled from plume metagenomes (SUP05-GB1 and SUP05-GB2) (48) and the two genomes of recent SUP05 isolates (*Candidatus Thioglobus singularis* and *Candidatus Thioglobus autotrophica* EF1) (45,46). The most similar host for the Lau220 genomes was the assembled host genome SUP05-GB1 (Table 5). The consensus among the top five most similar hosts at the class level was correct (Gammaproteobacteria) for the Lau77 and Lau220 genomes, but for the remaining viruses, the consensus prediction was Flavobacteriia (phylum Bacteroidetes) (Table 5).

**Table 5.** Host predictions for 18 SUP05 metagenomically-assembled viruses

			Host taxonomy prediction using marine host genomes		Host taxonomy prediction using metagenomic host contigs	
Virus genome	Has sulfur genes?^a^	Virus type	Most similar host genome^b^	Class consensus (top 5)^c^	Most similar contig^d^	Consensus (top 5)^c^
Lau218_Abe	Y	Podovirus	*Flavobacterium sp*. ACAM 123	Flavobacteriia	bin SUP05-GB1	SUP05
Lau218_KiloMoana	Y	Podovirus	*Flavobacterium sp*. ACAM 123	Flavobacteriia	bin SUP05-GB1	SUP05
Lau218_TahiMoana	Y	Podovirus	*Flavobacterium sp*. ACAM 123	Flavobacteriia	bin SUP05-GB1	SUP05
Lau220_Abe	N	Unclassified	SUP05-GB1	γ*-*Proteobacteria	bin SUP05-GB1	SUP05
Lau220_KiloMoana	N	Unclassified	SUP05-GB1	γ-Proteobacteria	bin SUP05-GB1	SUP05
Lau220_TahiMoana	N	Unclassified	SUP05-GB1	γ-Proteobacteria	bin SUP05-GB1	SUP05
Lau77_Abe	Y	Myovirus	*Psychromonas sp*. SP041	γ-Proteobacteria	bin SUP05-GB1	SUP05
Lau77_KiloMoana	Y	Myovirus	*Psychromonas sp*. SP041	γ-Proteobacteria	bin SUP05-GB1	SUP05
Lau77_TahiMoana	Y	Myovirus	*Psychromonas sp*. SP041	γ-Proteobacteria	bin SUP05-GB1	SUP05
Lau85_Abe	Y	Myovirus	*F. sasangense* DSM 21067	Flavobacteriia	bin SUP05-GB2	SUP05
Lau85_Guaymas	Y	Myovirus	*F. sasangense* DSM 21067	Flavobacteriia	bin SUP05-GB1	SUP05
Lau85_KiloMoana	Y	Myovirus	*F. sasangense* DSM 21067	Flavobacteriia	bin SUP05-GB2	SUP05
Lau85_Mariner	Y	Myovirus	*F. sasangense* DSM 21067	Flavobacteriia	bin SUP05-GB2	SUP05
Lau85_TahiMoana	Y	Myovirus	*F. sasangense* DSM 21067	Flavobacteriia	bin SUP05-GB2	SUP05
Lau87_Abe	Y	Siphovirus	*F. indicum* DSM 17447	Flavobacteriia	bin SUP05-GB2	SUP05
Lau87_KiloMoana	Y	Siphovirus	*F. indicum* DSM 17447	Flavobacteriia	bin SUP05-GB2	SUP05
Lau87_Mariner	Y	Siphovirus	*F. indicum* DSM 17447	Flavobacteriia	bin SUP05-GB2	SUP05
Lau87_TahiMoana	Y	Siphovirus	*F. indicum* DSM 17447	Flavobacteriia	bin SUP05-GB2	SUP05

Predictions were made using $d_2^*$ (*k*-mer length 6) against 3,281 possible marine host genomes or 501 host contigs (≥5 kb) assembled from metagenomes from the Guaymas Basin hydrothermal vent plume and adjacent background seawater. No dissimilarity threshold was applied and predictions were made for all virus genomes.

^a^Virus genome possesses *rdsrA or rdsrC* sulfite oxidation genes.

^b^*F. sasangense* and *F. indicum* are *Flavobacterium* strains.

^c^Consensus of top five most similar hosts.

^d^The genomic bin to which the most similar contig belongs, named according to which assembled host genome, SUP05-GB1 or SUP05-GB2, belongs to that bin.

As a more relevant test of how the $d_2^*$ method might be applied in discovering novel virus-host interactions from metagenomic sequences, $d_2^*$ dissimilarities were computed between the 18 SUP05 viral genomes and metagenomic host contigs ≥5 kb assembled from both vent plume samples and nearby non-plume seawater. Host contigs belonged to SAR324 group bacteria (41%), SUP05 group bacteria (31%), members of the phylum *Thaumarchaeota* (16%), members of family *Methylococcales* (1%) and contigs of unknown taxonomy (11%). For all 18 viral genomes the most similar contig or consensus of the top five most similar hosts were consistently SUP05 group bacterial contigs, demonstrating the utility of ONF methods for correctly predicting virus-host interactions from metagenomes (Table 5). Correct prediction of host taxonomy for the 18 SUP05 genomes was independent of viral group and whether or not the genome contained sulfur oxidation genes. Interestingly, the viral genomes belonging to the Lau77, Lau218 and Lau220 were most similar to the SUP05-GB1 bin, and the remaining viruses were most frequently similar to the SUP05-GB2 bin, perhaps suggesting that these groups of viruses have different host ranges among SUP05 host populations.

crAssphage is a viral genome that was assembled from human fecal microbiome metagenomes, was found to be abundant and ubiquitous in humans, but whose true host is unknown (15). Using abundance profiling, Dutilh *et al*. (15) found that crAssphage abundance was strongly correlated to that of host genome sequences belonging to the genus *Bacteroides* in the bacterial phylum Bacteriodetes, and they inferred likely hosts of crAssphage belong to these taxa. We investigated host prediction results using the $d_2^*$ measure and *k*-mer length 6 of the query virus versus a focused set of 360 Human Microbiome Project (HMP) host genomes found in the human gut and used in the abundance profiling analysis in Dutilh *et al*. (15). Of these 360 hosts, the most similar strain (no dissimilarity threshold was applied) was a member of the genus *Coprobacillus* in the bacterial phylum Firmicutes. The most similar *Bacteroides* strain to crAssphage was ranked 57th in the 360 host dataset. Three genera, *Bacteroides, Coprobacillus* and *Fusobacterium*, out of 83 possible genera in the 360 HMP hosts were detected as having significantly lower dissimilarities than all other taxa using the rank-sum test analysis (Bonferroni corrected *P*-values = 1.64 × 10^−6^, 6.34 × 10^−3^ and 6.92 × 10^−6^). *Bacteroides* is the previously inferred host genus for crAssphage from the original study that assembled and discovered this virus. These three genera belong to the phyla Bacteroidetes, Firmicutes and Fusobacteria, respectively. Similarly, rank-sum analysis at the phylum level yielded only two phyla, Bacteroidetes and Fusobacteria, out of eight possible phyla.

## DISCUSSION

From previous studies, ONF based approaches have shown promise in predicting what host a particular virus infects by matching query viruses to the host with which they share the highest ONF similarity (14). Two major components impact the performance of these approaches—the type of measure and *k*-mer length used. A few studies have explored the impact of varying *k*-mer length for a given measure (9,14), but only one measure, Euclidean distance, has predominantly been implemented in ONF virus-host relationships (14,20). We therefore conducted a comprehensive comparison of 11 distance/dissimilarity measures over several *k*-mer lengths and using the largest set of benchmarking viruses tested to date: 1427 RefSeq virus genomes for which the host on which they were isolated is known. Of the measures tested, $d_2^*$ exhibited good performance in host prediction accuracy, especially at *k*-mer lengths 6 and 9 where it had the highest accuracies at most taxonomic levels. Previous studies have focused primarily on tetranucleotide measures, with some rightly cautioning that high *k*-mers result in sparse frequency profiles (frequency vectors with many zeroes) that may diminish their effectiveness in making predictions (14). We found that increasing *k*-mer length did diminish performance for some measures such as *Ma* and *Ch*, but for others including $d_2^*$ and *Eu*, stable increases in accuracy were observed. Our results overall show sizeable improvements in host prediction with $d_2^*$ in comparison to *Eu* distance. Because computational time increases 64 fold when moving from *k*-mer length 6 to 9, in practice we recommend use of *k*-mer length 6, unless computation time is not a factor.

$d_2^*$ has shown excellent performance in other applications for assessing relatedness of whole genomes or metagenomic samples (22,26–28), so perhaps it is not surprising that it was the top performing measure in virus-host prediction. $d_2^*$ along with other measures like $d_2^S$, *Hao* and *Teeling*, distinct from simpler measures like *Eu* and *Ma*, in that they take into consideration the background oligonucleotide patterns of the two sequences being compared. This background-subtracting feature of $d_2^*$ is likely why it produced superior host prediction, but interestingly the simple measure *Ma* performed better at several taxonomic levels at *k*-mer length 4 or only slightly worse at longer *k*-mers. One challenge in alignment-free ONF based sequence methods is that distantly related genomes may possess similar ONF patterns due to coincidental convergent evolution (see Introduction). It is possible that the superiority of $d_2^*$ lies in its ability to discriminate closely related genomes by detecting particular *k*-mer patterns that are specific to the closely related genomes. This hypothesis would require further in depth investigation that is beyond the scope and focus of this work.

Implementation of $d_2^*$ resulted in accuracies of 33% at the genus level to 75% at the phylum level when predicting the host taxonomy of the 1427 RefSeq viruses from among ∼32 000 possible bacterial and archaeal host genomes. These accuracies are considerably better than previous host prediction results reported by Edwards *et al*. when using Euclidean distance and *k*-mer length 4 (Figure 6). Our improved accuracy is notable in that the previous study used a more restrictive set of only 2,699 complete host genomes. In a direct comparison of our method using the same 820 virus and 2699 host genome database used in Edwards *et al.*, the $d_2^*$ measure not surprisingly produced considerably higher accuracies (25–99% relative improvement).

The Edwards *et al*. study more broadly compared host prediction methods, including the use of sequence homology between viruses and hosts (blastn, blastx, and exact matches of short words), matching of CRISPR sequences, virus-host co-variation, and *Eu*-based ONF methods. They found that homology searches of viruses against host genomes yielded the highest accuracies, for example ranging from 21% to 62% at the genus level (Table 4). Use of the $d_2^*$ method on this same virus and host dataset produced accuracies within the range of these homology methods (Table 4). Furthermore, when implementing consensus methods and requiring a dissimilarity threshold for prediction (see Discussion below), prediction using $d_2^*$ was comparable or exceeded all other methods (Table 4, Figure 6). This did however come at the cost of being able to make predictions on only half of the viruses.

In addition to higher accuracies, our improved ONF method has a potential advantage over homology-based methods for predicting the host of metagenomic viral contigs. Homology-based prediction methods require high sequence similarity of a diagnostic gene or sequence element (e.g. CRISPR or integration site) in the query virus to host. While both ONF and homology searches are host database dependent, fragmented metagenomic viral or host contigs may lack the specific gene(s) or element(s) that allow homology searches to infer their hosts. Alignment-free ONF methods however do not require a specific gene to make a prediction, and for this reason may have an advantage over homology-based methods. Consistent with this, subsampling host genomes even at 10 kb did not appreciably diminish prediction accuracy (Figure 3A). We also show that host prediction is insensitive to sequencing error at rates ≤ 0.01 (Figure 3B). Given that the upper bound of reported error rates for Illumina and 454 sequencing technology is 0.01 (40), our simulation results indicate that our *k*-mer based method $d_2^*$ applied to metagenomic samples is not strongly impacted by sequencing errors.

While genus level accuracies of ∼30–40% are rather low for a predictive tool, higher taxonomic levels had higher host prediction accuracies, reaching 60–70% at the class and phylum levels. There are a large number of bacterial and archaeal phyla for which viruses that infect them have yet to be identified (9). There is a pressing need to identify hosts at this broad level, thus given its great accuracy at the phylum level, the $d_2^*$ ONF method has potential to meaningfully advance our characterization of novel viruses.

Previous work by Roux *et al*. (21) using ONF matching methods (with Manhattan distance and *k*-mer length 4) curiously reported much higher accuracies than found here (60-90% at the genus to order levels without thresholding). Direct comparison of the measures *Ma* and $d_2^*$ for their and our virus and host datasets confirmed higher accuracies with their dataset, often two-fold higher (Figure 7). We suspect this is because of a fundamental difference in virus and host datasets and the resulting composition of viruses being tested. The Roux *et al*. dataset consists of sequences they report as viruses that were identified from within host genome sequencing projects using their virus finding software VirSorter (9). Considering these viruses are generally from host cells, they probably contain many proviruses that are integrated into the host genome, as well as non-integrating viruses that stably exist within the cell for long periods such as ‘extrachromosomal prophages’ (or ‘plasmid prophage’) (49) or viruses with ‘carrier’ or ‘chronic’ cycles (50,51). Most of viruses in our 1427 isolate database, in contrast, are likely to be predominantly lytic and only exist briefly in the host cell during infection. Also, they are all ‘bona fide’ viruses based on isolation from cultures rather than sequences characterized as viruses based on sequence matching. Proviruses and other viruses that stably co-exist in the host cell are hypothesized to experience a higher degree of ONF amelioration than lytic viruses (52). This presumed difference in the proportion of viruses of different life-styles in these two datasets and potential artifacts and circularity in the viral sequences used in the Roux *et al*. study probably explains the marked difference in prediction accuracies. Irrespective of what exactly explains the difference, we suggest that our results provide a more realistic expectation of prediction accuracy using ONF methods on metagenomic viral contigs, something which has not be implemented by previous virus-host matching studies.

A few approaches were used to further increase host prediction accuracy with the $d_2^*$ measure. Using a consensus approach for selecting the predicted host rather than simply choosing the host with the absolute lowest dissimilarity helped improve host prediction across all taxonomic levels (Figure 4A). Adopting a method employed by Roux *et al*. (21), requiring a maximum dissimilarity threshold for making a host prediction also improved host prediction; however it came at the cost of reducing the number of viruses for which predictions could be made (Figure 4B). Combination of both methods further increased prediction accuracy while helping increase the number of taxa for which predictions could be made (Figure 4C).

Although these methods can improve host prediction accuracy, there are still some important limitations to consider with any ONF-based method. First, a close relative to the true host of the query virus may not be present in host database. This should become less of an issue as sampling of host genome diversity continues to grow rapidly and as explained above, or when using host metagenomic contigs in the analysis. Second, it is always possible that some viruses just may not exhibit high ONF similarity to its host. Indeed, there was a broad range in virus-host distance/dissimilarity among known virus-host pairs (Figure 1). Another important finding of this study is that the degree of virus-host distance/dissimilarity varies between virus groups and reflects fundamental differences in their biology. Within the group of tailed caudoviruses, myoviruses exhibited considerably higher distance/dissimilarity to their hosts than podoviruses and siphoviruses (Figure 8). In a few studies, myoviruses are observed to have broader host ranges than the other groups and siphoviruses often have the narrowest host ranges (41,42). Thus virus-host distance/dissimilarity appears to generally relate positively to host range. It is unclear on an evolutionary mechanistic level whether host range drives virus-host distance/dissimilarity, vice versa, or if it involves a balanced interplay in both directions. In practical terms for host prediction methods, these results indicate that host predictions on siphoviruses can be made with higher confidence.

A third complication in ONF methods is that some viruses may coincidentally have low distance/dissimilarity to hosts that they do not actually infect. As mentioned above, distantly related hosts and viruses may possess similar oligonucleotide patterns via independent, convergent evolution. In terms of host prediction, this means that the true host may be interspersed among other non-specific hosts, making it difficult to make accurate host predictions. Rank-sum test analysis was implemented to partially mitigate this issue by detecting particular taxa that have significantly lower dissimilarities than all other taxa (*P* < 0.05). In the case of crAssphage, this rank-sum test approach did predict the same host genus (*Bacteroides*) phylum (Bacteroidetes) as the previously inferred host phylum by co-variance methods (15), whereas the simpler methods of finding the absolute lowest dissimilar host did not. One limitation of the rank-sum approach is that if very few members of the true host exist in the database, rank-sum analysis will not have enough statistical power to predict that host even if they have lower dissimilarities. Host prediction results on crAssphage also highlight how the $d_2^*$ method can be useful for hypothesis generation. It is generally recognized that viruses rarely infect hosts from multiple phyla, but a few cases have been reported (53). The $d_2^*$ method predicted two possible host phyla for crAssphage, Bacteroidetes and Fusobacteria, which could be tested directly if and when crAssphage viruses are isolated.

Another solution to improving prediction accuracy and to potentially limit non-specific hosts is to only look among those that share the same habitat. This approach increased host prediction accuracy using $d_2^*$ for marine viruses when the possible hosts were restricted to only those that are marine, and we expect that this would be the case for any reasonable measure used. We implemented this type of host limitation in our host prediction of crAssphage by only using 360 relevant gut microbe genomes as possible hosts. Rank-sum analysis in particular suggest that the host of crAssphage belongs to the genera *Bacteroides, Coprobacillus* and *Fusobacterium*, the former of which was previously inferred host genus based on abundance co-variation (15).

For making predictions on metagenomically assembled viruses, one similar, promising approach in limiting relevant host sequences is to use metagenomic host contigs from the same samples or similar sample types as those of the viruses. To our knowledge, this is the first reported use of ONF methods for virus-host prediction using metagenomic sequences. This approach yielded excellent results in our analysis of SUP05 viruses assembled from vent plume metagenomes. When comparing these viruses to marine hosts from NCBI, some of the viruses could be correctly predicted at the class or phylum level, but when dissimilarities were computed to only host contigs from vent plume metagenomes, the $d_2^*$ method achieved perfect host prediction (assuming that the inferred host of these viruses based on the presence of host sulfur oxidation genes is correct) (Table 5). In the specific case of the Lau220 viruses, for which the sulfur oxidation genes are absent, we were able to predict its host taxonomy. Furthermore, our host prediction results suggest that particular SUP05 viruses may infect particular subgroups of SUP05 hosts. We strongly recommend this approach in future host prediction studies on metagenomic viral contigs.

Metagenomic assembly often yields partial genomes, so host prediction using subsampled sequences from the 1427 complete viral genomes was used to assess the dependence of prediction accuracy on the length of the query viral contig. Host prediction was not grossly compromised when subsampling at lengths of 10 kb and above (< 11% drop in accuracy compared to full length genomes). While prediction accuracy began to drop more markedly for smaller contigs, it may still be feasible to make decent predictions on contigs of 5 kb and above. For reference, the metgenomically assembled crAssphage and SUP05 viral genomes were much greater than 20 kb (crAssphage: 97 kb; SUP05: 39–333 kb). In the case of predicting the host of viral contigs from metagenomic host contigs, it may be necessary to further assess how accuracy is impacted by both viral and host contig lengths as well as determine relevant threshold levels for $d_2^*$ scores for implementing the thresholding approach.

This study provides a comprehensive assessment of various distance/dissimilarity measures for use in host prediction methods and specifically recommends the $d_2^*$ measure for optimum prediction accuracy. The $d_2^*$ measure along with improvements in selecting the predicted host yielded comparable or better prediction accuracies than previous top performing homology-based methods. We suggest ONF methods used in conjunction with abundance profiling and homology methods will provide a powerful and improved approach for predict the hosts of novel viruses recovered from metagenomes. Congruent, independent host prediction by multiple methods could provide a means of validation on the accuracy of the prediction. Case in point is congruent host predictions for crAssphage by the $d_2^*$ method and the original host prediction based on an abundance profiling approach. We also have provided a simple program entitled VirHostMatcher for computing ONF scores and visualization of results. With this improved method, there is an exciting opportunity to extensively expand our knowledge of viral diversity and virus-host interactions using the rapidly increasing metagenomic datasets available from all types of microbial habitats (11,12,15,54).
